# The Effect of Optical Crosstalk on Accuracy of Reflectance-Type Pulse Oximeter for Mobile Healthcare

**DOI:** 10.1155/2018/3521738

**Published:** 2018-10-21

**Authors:** Hyun Jae Baek, JaeWook Shin, Jaegeol Cho

**Affiliations:** Department of Medical and Mechatronics Engineering, Soonchunhyang University, Asan, Chungnam, Republic of Korea

## Abstract

According to the theoretical equation of the pulse oximeter expressed by the ratio of amplitude (AC) and baseline (DC) obtained from the photoplethysmographic signal of two wavelengths, the difference of the amount of light absorbed depending on the melanin indicating the skin color is canceled by normalizing the AC value to the DC value of each wavelength. Therefore, theoretically, skin color does not affect the accuracy of oxygen saturation measurement. However, if there is a direct path for the light emitting unit to the light receiving unit instead of passing through the human body, the amount of light reflected by the surface of the skin changes depending on the color of the skin. As a result, the amount of crosstalk that varies depending on the skin color affects the ratio of AC to DC, resulting in errors in the calculation of the oxygen saturation value. We made crosstalk sensors and crosstalk-free sensors and performed desaturation experiments with respiratory gas control on subjects with various skin colors to perform oxygen saturation measurements ranging from 60 to 100%. Experimental results showed that there was no difference in the measurement error of oxygen saturation according to skin color in the case of the sensor which prevented crosstalk (−0.8824 ± 2.2859 for Asian subjects, 0.6741 ± 3.2822 for Caucasian subjects, and 0.9669 ± 2.2268 for African American subjects). However, a sensor that did not prevent crosstalk showed a large error in dark skin subjects (0.8258 ± 2.1603 for Asian subjects, 0.8733 ± 1.9716 for Caucasian subjects, and −3.0591 ± 3.9925 for African Americans). Based on these results, we reiterate the importance of sensor design in the development of pulse oximeters using reflectance-type sensors.

## 1. Introduction

**Figure 1:**
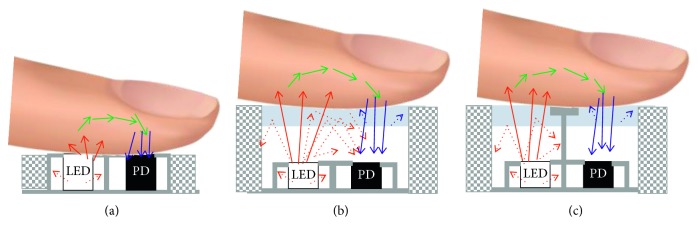
Examples of reflectance-type pulse oximetry sensors.

Since the Japanese biomedical engineer, Takuo Aoyagi first proposed the idea of using pulsatile light variation to measure arterial oxygen saturation in the 1970s, a variety of pulse oximeters have been studied and developed in many universities and research institutes around the world [1, 2]. Pulse oximeters, which are currently used in medical facilities for patient monitoring in the intensive care unit, and anesthesia in the operating room are most often used with finger clip type sensors. This can be regarded as a transmittance-type sensor because the light emitting portion for measuring the oxygen saturation is on one side of the body and the light receiving portion is located on the opposite side. Because of these morphological features, the transmittance-type sensor is mainly used for finger or toe. On the other hand, since the reflectance-type sensor has both the light emitting portion and the light receiving portion on the same plane, it can be applied to various body parts, and a representative example is forehead pulse oximeter. Reflectance-type sensors have not been short in their history and have been developed in a variety of forms, but they are still not widely used in practice compared to transmittance-type sensors for many reasons [3, 4]. When a transmittance-type sensor is used for a recording of pulsation, it can be measured ten times more strongly than when a reflection type sensor is used [5]. In addition, since the sensor is well fixed to the finger in the form of a clip, the possibility of movement of the sensor and the possibility of entrance of external light are small, so that the quality of the signal to be measured is much more prevalent. On the other hand, in the case of the reflectance-type sensor, if the sensor is not fixed with the adhesive, the sensor slips over the skin, resulting in motion noise. Nevertheless, as the mobile health devices have recently been attracting attention, there is a growing demand for pulse oximeters using reflectance-type sensors [6]. As a mobile healthcare device, modern reflectance-type pulse oximeter has led to changes in optical sensor configurations. Conventional reflectance-type pulse oximetry sensors consist of LEDs with two or more wavelengths and a photodetector, which are spaced 4–11 mm apart [7]. As shown in Figure 1, an optical barrier is required to prevent direct coupling between the light emitting part and the light receiving part and is applied in the form of a partition between the LED and the photodetector in the optical sensor. Typically, LEDs and photodetectors are packaged and physically separated using a black rubber material. LEDs and photodetector are exposed on the surface of the sensor or coated with transparent epoxy. In both cases, LEDs and photodetector, which are completely separated, are in direct contact with the skin. As a result, the photon irradiated from the light emitting part can reach the light receiving part only through the human body. In recent years, optical sensor technology for pulsation measurement has become popular, and the LED-photodetector pair is integrated with the analog front-end, making it miniaturized and becoming a single component. For example, Maxim's MAX30100 includes both optical sensors and measurement modules in a small size of 5.6 × 2.8 × 1.2 mm [8]. Although the sensor module itself is completely physically separated from the light emitting unit and the light receiving unit, a gap is formed between the sensor module and the device while the module is mounted on the device. Also, in some cases, there is also cross talk caused by cover grass, as described in Figure 1. As a result, unlike conventional sensors, photons directly coupled to the inside of the sensor or reflected from the skin surface without passing through the human body are measured together at the light receiving part of pulse oximetry sensor.

In this paper, we emphasize the importance of sensor design to prevent crosstalk when using a small modular reflectance-type pulse oximetry sensor according to recent trends. Specifically, we analyzed the effect of optical crosstalk on the reflectance-type pulse oximeter according to the subject's skin color through theoretical and clinical studies. First, the Beer–Lambert law, the theoretical background of pulse oximetry, was analyzed. Then, desaturation test was performed to reduce the oxygen saturation to 70% by controlling the respiratory gas, and the oxygen saturation measurement results were compared using the sensors with and without crosstalk prevention. Desaturation testing was approved by the Shenzhen University Committee on Human Research and was conducted with the consent of all subjects.

## 2. Theoretical Formulation

The principle of the pulse oximeter can be explained by modified Beer–Lambert law [2, 9, 10], where, *I*(*λ*) is detected light intensity, *I*_*o*_(*λ*) is the incident light intensity, *ε*(*λ*) is a molar absorption coefficient, *C* is the molar concentration, $\bar{l}\left(\lambda \right)$ is them mean path length, and *G*(*λ*) is appropriate factors that account for the measurement geometry. The signal that records changes in *I*(*λ*) caused by pulsatile cardiac activity is called photoplethysmogram (PPG). If we assume that the light absorbing materials are melanin and other blood or skin-related components, the amplitude for the red wavelength PPG signal (AC) can be expressed as Equation (2). The subscripts m and b denote melanin and blood, and *A*_0_ is a spectrum of all chromophores in human skin except m and b. Also, the baseline (DC) can be written as

(1) $$\begin{array}{c} A\left(\lambda \;\right)=\ln\frac{I_{o}\left(\lambda \right)}{I\left(\lambda \right)}=\varepsilon \left(\lambda \right)C\bar{l}\left(\lambda ,C\right)+G\left(\lambda \right), \end{array}$$

(2) $$\begin{array}{c} {\text{AC}}_{\text{Red}}=I_{0}\left(\lambda_{\text{Red}}\right)\exp\left[-\varepsilon_{\text{m}}\left(\lambda_{\text{Red}}\right)C_{\text{m}}{\bar{l}}_{\text{m}}\left(\lambda_{\text{Red}}\right)-\varepsilon_{\text{b}}\left(\lambda_{\text{Red}}\right)C_{\text{b}}{\bar{l}}_{\text{b}-\text{dia}}\left(\lambda_{\text{Red}}\right)-A_{0}\left(\lambda_{\text{Red}}\right)\right]-I_{0}\left(\lambda_{\text{Red}}\right)\exp\left[-\varepsilon_{\text{m}}\left(\lambda_{\text{Red}}\right)C_{\text{m}}{\bar{l}}_{\text{m}}\left(\lambda_{\text{Red}}\right)-\varepsilon_{b}\left(\lambda_{\text{Red}}\right)C_{\text{b}}{\bar{l}}_{\text{b}-\text{sys}}\left(\lambda_{\text{Red}}\right)-A_{0}\left(\lambda_{\text{Red}}\right)\right]\;\;\mathrm{where},\;\;A_{0}\left(\lambda_{\text{Red}}\right)=A_{0}^{\prime }\left(\lambda_{\text{Red}}\right)+G\left(\lambda_{\text{Red}}\right), \end{array}$$

(3) $$\begin{array}{c} {\text{DC}}_{\text{Red}}=I_{0}\left(\lambda_{\text{Red}}\right)\exp\left[-\varepsilon_{\text{m}}\left(\lambda_{\text{Red}}\right)C_{\text{m}}{\bar{l}}_{\text{m}}\left(\lambda_{\text{Red}}\right)-\varepsilon_{\text{b}}\left(\lambda_{\text{Red}}\right)C_{\text{b}}{\bar{l}}_{\text{b}-\text{sys}}\left(\lambda_{\text{Red}}\right)-A_{0}\left(\lambda_{\text{Red}}\right)\right]. \end{array}$$

The ratio of the AC to the DC reflects the change in the maximum blood volume during the systolic period (MBVSYS). The oxygen saturation can be obtained by using the ratio of MBVSYS for the infrared to those for the red since the red light is less absorbed in HbO_2_ than in Hb and on the contrary in the case of infrared. From (2) and (3), MBVSYS for the red and infrared wavelength PPG can be obtained as Equations (4) and (5), respectively, where $\alpha \left(\lambda \right)=-\varepsilon_{\text{m}}\left(\lambda \right)C_{\text{m}}{\bar{l}}_{\text{m}}\left(\lambda \right)$, $\beta \left(\lambda \right)=-\varepsilon_{\text{b}}\left(\lambda \right)C_{\text{b}}{\bar{l}}_{\text{b}}\left(\lambda \right)$, and *γ*(*λ*)=−*A*_0_(*λ*). Finally, the ratio for the oxygen saturation calculation can be derived as *R*=[AC_Red_/DC_Red_]/[AC_IR_/DC_IR_] and is linked to the oxygen saturation in a function of SpO_2_=*f*(*R*).

(4) $$\begin{array}{c} \frac{{\text{AC}}_{\text{Red}}}{{\text{DC}}_{\text{Red}}}=\frac{I_{0}\left(\lambda_{\text{Red}}\right)\exp\left[\alpha \left(\lambda_{\text{Red}}\right)+\beta_{\text{dia}}\left(\lambda_{\text{Red}}\right)+\gamma \left(\lambda_{\text{Red}}\right)\right]-I_{0}\left(\lambda_{\text{Red}}\right)\exp\left[\alpha \left(\lambda_{\text{Red}}\right)+\beta_{\text{sys}}\left(\lambda_{\text{Red}}\right)+\gamma \left(\lambda_{\text{Red}}\right)\right]}{I_{0}\left(\lambda_{\text{Red}}\right)\exp\left[\alpha \left(\lambda_{\text{Red}}\right)+\beta_{\text{sys}}\left(\lambda_{\text{Red}}\right)+\gamma \left(\lambda_{\text{Red}}\right)\right]}=\frac{\exp\left[-\varepsilon_{\text{b}}\left(\lambda_{\text{Red}}\right)C_{\text{b}}{\bar{l}}_{\text{b}-\text{dia}}\left(\lambda_{\text{Red}}\right)\right]-\exp\left[-\varepsilon_{\text{b}}\left(\lambda_{\text{Red}}\right)C_{\text{b}}{\bar{l}}_{\text{b}-\text{sys}}\left(\lambda_{\text{Red}}\right)\right]}{\exp\left[-\varepsilon_{\text{b}}\left(\lambda_{\text{Red}}\right)C_{\text{b}}{\bar{l}}_{\text{b}-\text{sys}}\left(\lambda_{\text{Red}}\right)\right]}≅\varepsilon_{\text{b}}\left(\lambda_{\text{Red}}\right)C_{\text{b}}\left[{\bar{l}}_{\text{b}-\text{sys}}\left(\lambda_{\text{Red}}\right)-{\bar{l}}_{\text{b}-\text{dia}}\left(\lambda_{\text{Red}}\right)\right], \end{array}$$

(5) $$\begin{array}{c} \frac{{\text{AC}}_{\text{IR}}}{{\text{DC}}_{\text{IR}}}≅\varepsilon_{\text{b}}\left(\lambda_{\text{IR}}\right)C_{\text{b}}\left[{\bar{l}}_{\text{b}-\text{sys}}\left(\lambda_{\text{IR}}\right)-{\bar{l}}_{\text{b}-\text{dia}}\left(\lambda_{\text{IR}}\right)\right]. \end{array}$$

If there is crosstalk in pulse oximetry sensor like Figure 1, detected light intensity will be *I*(*λ*)+*I*_c_(*λ*), and the MBVSYS for ref PPG is rewritten as Equation (6), where subscript c denotes crosstalk. As described in (7), *I*_c_(*λ*) makes a change in the ratio value, which again causes an oxygen saturation error.

(6) $$\begin{array}{c} \frac{{\text{AC}}_{\text{Red}}^{\prime }}{{\text{DC}}_{\text{Red}}^{\prime }}=\frac{\left\{I_{0}\left(\lambda_{\text{Red}}\right)\exp\left[\alpha \left(\lambda_{\text{Red}}\right)+\beta_{\text{dia}}\left(\lambda_{\text{Red}}\right)+\gamma \left(\lambda_{\text{Red}}\right)\right]+I_{\text{c}}\left(\lambda_{\text{Red}}\right)\right\}\;-\;\left\{I_{0}\left(\lambda_{\text{Red}}\right)\exp\left[\alpha \left(\lambda_{\text{Red}}\right)+\beta_{\text{sys}}\left(\lambda_{\text{Red}}\right)+\gamma \left(\lambda_{\text{Red}}\right)\right]+I_{\text{c}}\left(\lambda_{\text{Red}}\right)\right\}}{I_{0}\left(\lambda_{\text{Red}}\right)\exp\left[\alpha \left(\lambda_{\text{Red}}\right)+\beta_{sys}\left(\lambda_{\text{Red}}\right)+\gamma \left(\lambda_{\text{Red}}\right)\right]+I_{\text{c}}\left(\lambda_{\text{Red}}\right)}. \end{array}$$

(7) $$\begin{array}{c} \frac{{\text{AC}}_{\text{Red}}^{\prime }}{{\text{DC}}_{\text{Red}}^{\prime }}\leq \frac{{\text{AC}}_{\text{Red}}}{{\text{DC}}_{\text{Red}}},\;\;R^{\prime }=\frac{{\text{AC}}_{\text{Red}}^{\prime }/{\text{DC}}_{\text{Red}}^{\prime }}{{\text{AC}}_{\text{IR}}^{\prime }/{\text{DC}}_{\text{IR}}^{\prime }}\neq \frac{{\text{AC}}_{\text{Red}}/{\text{DC}}_{\text{Red}}}{{\text{AC}}_{\text{IR}}/{\text{DC}}_{\text{IR}}}. \end{array}$$

## 3. Experiments

**Figure 2:**
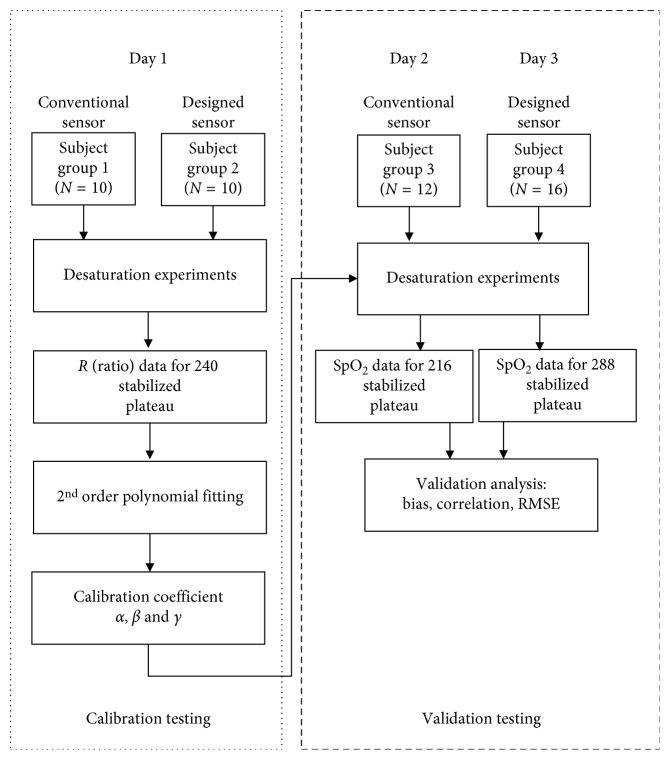
Flowchart of the experimental procedure.

**Figure 3:**
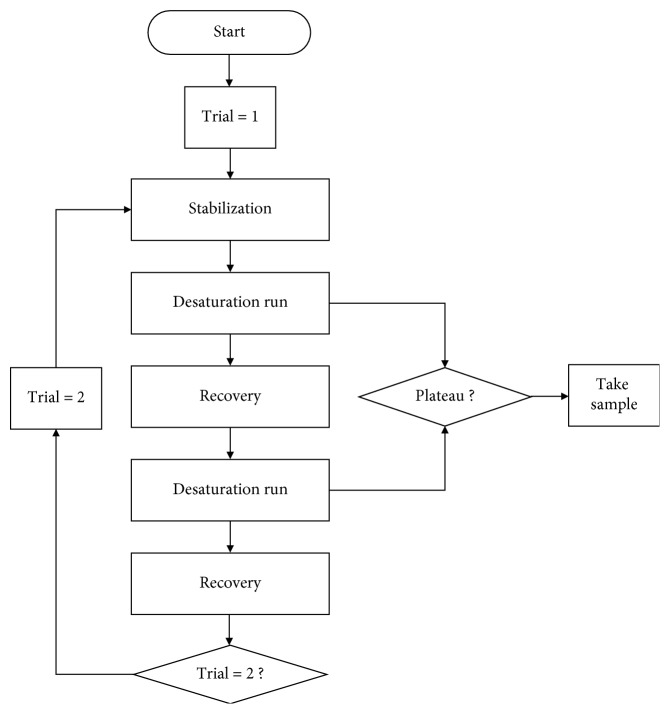
Schematic flow for the desaturation testing.

We refer to the sensor described in Figure 1 as the conventional sensor and the sensor described in Figure 1 as the designed sensor. The optical sensor module MAX30100 (Maxim Integrated Products, Inc., San Jose, CA) was employed for both sensors [8]. It combines two LEDs with a typical wavelength of 880 nm and 660 nm, photodetector, optimized optics, and 16-bit sigma-delta analog-to-digital converter. In addition, it has function for ambient light cancellation and adjustable digital filter to reject power interference and ambient noise. In order to control the MAX30100 system including LED drive circuit, we used the Cortex M4, STM32F401 (STMicroelectronics, Dallas, TX, USA), which offers the balance of dynamic power consumption and processing performance. The only difference was that the designed sensor shown in Figure 1 employed a barrier structure for physically separating the LED and photodetector on the cover glass. The experimental flowchart is shown in Figure 2. Two desaturation experiments were conducted for each sensor: (1) calibration testing and (2) validation testing. For each subject and each experiment, a series of desaturation runs were performed as shown in Figure 3. Each run started with a stabilized period at room air and is followed by stabilized plateaus at various lower saturation levels between 60 and 100%. The following were the value targets: Run1—92%, 87%, 82%, 77%, and 72% and Run2—93%, 88%, 83%, 78%, and 73%. The objective of these targets was to spread the data points evenly over the desired range. Achieving them exactly was not important. Though every effort was made to be within 2%. A plateau was defined as stable when the readings of the reference pulse oximeter manufactured by Nellcor oximeter (N-550, Nellcor Puritan Bennett Inc., Pleasanton, CA) have not changed more than 1% for 10 seconds.

For each subject, this allows 24 stabilized plateaus to be taken when initial room air saturation is considered (series of desaturation runs: Run1-Run1 and Run2-Run2). Therefore, for example, 10 subjects produce 240 stabilized plateaus. Reducing oxygen saturation level was performed by a skilled operator at Shenzhen University, China, by adjusting the inspired air-nitrogen-CO_2_ mixture for the SaO_2_ response predicted from the oxyhemoglobin dissociation curve obtained by end tidal gas analysis [11]. A common sense is that dark skinned subjects are known to have a palm area that is not darker than the skin color of other parts of the body. But even so, recent studies have shown that they have a darker color than the palm colors of Asian and Caucasian [12]. Based on this, we measured the PPG signals from the finger to investigate the effect of optical crosstalk on the pulse oximeter according to skin color. Before starting the experiment, we attached a sensor to the index finger of the subject and fixed it with adhesive tape so that it would not move because of over breathing. During the measurement of the data, the subjects were placed in a relaxed semisupine posture with a mouthpiece for breathing in a chair about 30 degrees inclined.

### 3.1. Calibration Testing

The ratio of absorbencies at two wavelengths value (*R*=[AC_Red_/DC_Red_]/[AC_IR_/DC_IR_]) was calibrated empirically against reference oxygen saturation measured by N-550 pulse oximeter manufactured by Nellcor (Nellcor Puritan Bennett Inc., Pleasanton, CA) in volunteers. To relate the measured values of the ratio *R* to the oxygen saturation reading of the pulse oximeter, the empirical calibration curve was derived by a second order polynomial, SpO_2_=*α*+*β* · *R*+*γ* · *R*^2^. The coefficients, *α*, *β*, and *γ* were determined by regression analysis to give the curve a best fit to the reference SpO_2_ for each sensor type. 10 subjects were recruited for each calibration test, of which 3 were African American with dark skin color, and the rest were Caucasian or Asian. Therefore, total 240 stabilized plateaus for each sensor type were used for calibration.

### 3.2. Validation Testing

On the other day, the second experiment was performed to evaluate the accuracy of the oxygen saturation value obtained through the equation derived by calibration testing. For the conventional sensor shown in Figure 1, total 12 subjects were recruited and 3 of them were subjects with dark skin. This produces 216 stabilized plateaus for Caucasian or Asian and 72 stabilized plateaus for African American. Also, 16 were recruited for the designed sensor shown in Figure 1, and 4 of them were dark skinned subjects (288 stabilized plateaus for Caucasian or Asian and 96 stabilized plateaus for African American). Comparison of calibrated pulse oximetry with reference pulse oximeter measurements (N-550, Nellcor Puritan Bennett Inc., Pleasanton, CA) was reported in terms of the correlation coefficients and root mean squared error. Also, Bland–Altman plot was used to evaluate the discrepancy between measurements. Finally, the bias (reference SpO_2_—estimated SpO_2_ using calibrated equation) ± precision (standard deviation) of the oximeters was investigated according to different ranges of oxygen saturation.

## 4. Result

**Figure 4:**
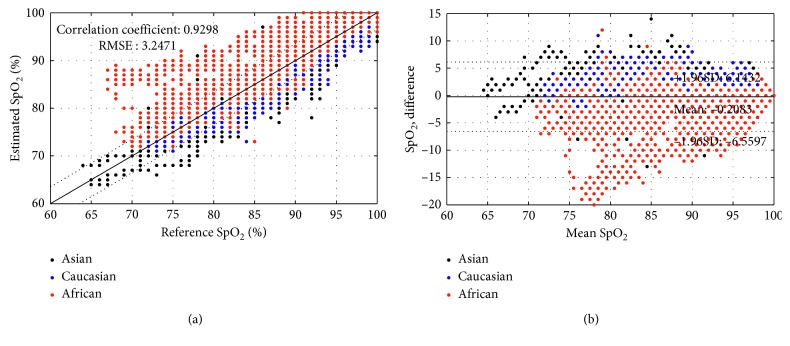
Accuracy of oxygen saturation estimation in a conventional sensor with optical crosstalk: (a) correlation plot; (b) Bland–Altman plot.

**Figure 5:**
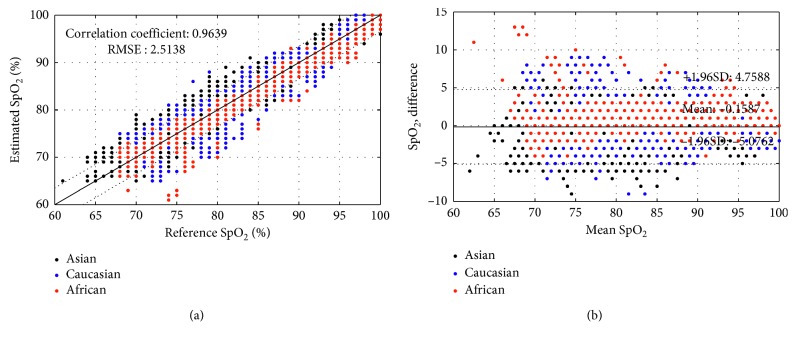
Accuracy of oxygen saturation estimation in a designed sensor without optical crosstalk: (a) correlation plot; (b) Bland–Altman plot.

Figures 4 and 5 show correlation, and Bland–Altman plots describe the accuracy results for the sensors shown in Figures 1 and 1, respectively. For the sensor shown in Figure 1 with crosstalk (conventional sensor), the oxygen saturation estimation error was −0.2083 ± 3.2405 for all subjects. The error by race was 0.8258 ± 2.1603 for Asian subjects, 0.8733 ± 1.9716 for Caucasian subjects, and −3.0591 ± 3.9925 for African Americans. On the other hand, in the absence of crosstalk (designed sensor), there was no difference in estimation error according to skin color as shown in Figure 3. The estimation error of oxygen saturation was −0.1587 ± 2.5089 for all subjects, −0.8824 ± 2.2859 for Asian subjects, 0.6741 ± 3.2822 for Caucasian subjects, and 0.9669 ± 2.2268 for African American subjects. The correlation coefficient between the reference oxygen saturation and the estimated value was 0.9298 when measured with conventional sensor and 0.9639 when measured with designed sensor for all subjects. By subject race, the correlation coefficient was 0.8864 for the results of using the conventional sensor (with crosstalk) for African American subjects and 0.94 or higher for all except the case.

**Figure 6:**
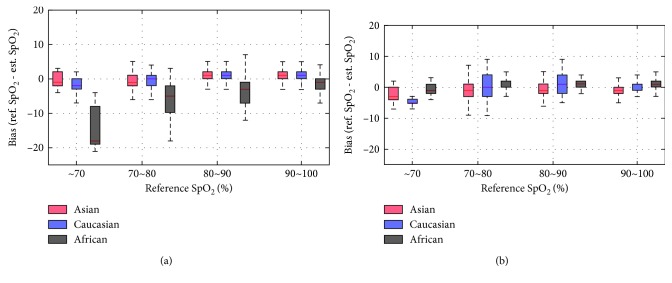
Boxplot of bias for the three different subject groups in different ranges of oxyhemoglobin saturation. (a) Conventional Sensor. (b) Designed Sensor.

In Figure 6, the boxplot shows median centerline, the 1st and 3rd quartile (box outline), and minimum and maximum values (whiskers) of SpO_2_ estimation error in different ranges of oxygen saturation. The presence of crosstalk in the reflectance-type pulse oximetry sensor (conventional sensor) tended to overestimate overall oxygen saturation in subjects with dark skin, and the estimation error was particularly large at low oxygen saturation.  Table 1 shows the bias between reference and estimated SpO_2_ according to race (skin color) and sensor type. At the SpO_2_ level of less than 70%, the largest error of −15.05 ± 5.58% was found in the African American subject group when measured using conventional sensors. Even at 80–90% oxygen saturation levels of African Americans measured with conventional sensors, they showed a greater level of error than in all other cases. There was no difference in the measurement accuracy of oxygen saturation according to the race in the case of the designed sensor which blocked the optical crosstalk.

## 5. Discussion and Conclusion

Since the pulse oximetry technology is already well known and can be implemented easily, it has been recently adopted by general consumer device makers rather than medical device companies to develop mobile healthcare products. There are, however, few studies on reflectance-type pulse oximetry sensors that are used primarily for this purpose. Recent published wearable pulse oximeter studies have used reflectance-type sensors, but most of them have not been tested for subjects with various skin colors, especially dark skin color. Lu et al. proposed a reflection pulse oximeter embedded in the back cover of a smart handheld device [13]. SpO_2_ was tested in the range of 80–100% for 16 subjects, but there was no skin color information of the subjects. In these prototypes, optical crosstalk between the back cover and the main board on which the LED and PD were mounted was not blocked, so it is expected that errors may occur in dark skin subjects, especially in SpO_2_ which is lower than 80%. Poh et al. proposed an earphone-type reflective PPG measurement system and suggested that oxygen saturation can be measured by adding LED of different wavelength [14]. LED and phototransistor in their prototypes were integrated into a small resin package and mounted on the earbud of the earphone. Therefore, although the experimental results on oxygen saturation are not presented, if the optical barrier design is not carefully illustrated as shown in Figure 1, SpO_2_ measurement error is likely to occur in dark skin. Venema et al. also proposed a similar system [15]. They named it in-ear pulse oximetry because the prototype optical sensor was placed at the inner tragus. They sealed an optical sensor into an ear mold. They have customized the ear mold to the individual, but careful design is needed to ensure that the surface of the optical sensor is in perfect contact with the skin surface of the tragus to prevent direct crosstalk. They showed the result of sudden SpO_2_ drop due to sleep apnea during sleep, but there was no measurement of dark skin subject. Recently, studies have been actively conducted to measure PPG signals in a noncontact manner using a web camera or a smartphone camera [16–18]. It enables noncontact heart rate monitoring by detecting cardiac pulse induced subtle color variation on skin surface. Especially, in recent years, it has been tried to measure not only heart rate but also oxygen saturation using camera [19–22]. Most studies have been performed about low-sampling rate (frame rate), noise due to ambient light, and light source for metrology, and crosstalk in noncontact situations was simply overlooked as one of the noise sources. However, it is expected that an error due to skin color will occur when camera is used for pulse oximetry, so it is necessary to test subjects having various skin colors.

In this paper, we reviewed the importance of sensor design to prevent direct crosstalk in reflectance-type pulse oximetry sensor through theoretical analysis and experiments. According to the theoretical equation of the pulse oximeter expressed by the ratio of AC and DC obtained from the PPG signal of two wavelengths, the difference of the amount of light absorption depending on the melanin indicating the skin color is canceled by normalizing the AC value to the DC value of each wavelength. In other words, it can be explained that the term including the subscript “m” in the ratio formula of AC and DC shown in Equation (4) is canceled in the final formula. However, if the crosstalk occurs as in the case of the sensor shown in Figure 1, there is a term represented by a subscript “c” of different values depending on the color of the skin. Therefore, the presence of crosstalks increases the probability of error in the measurement of oxygen saturation depending on the skin color. Experimental results also show that most of the errors in oxygen saturation in conventional sensors suffer from optical crosstalks occurred in dark skin subjects. In conclusion, optical barrier design to prevent direct light crosstalk is critical to ensure accuracy regardless of skin color in reflective-type pulse oximeters for mobile healthcare devices.

## Figures and Tables

**Table 1:** Bias, reference SpO_2_ minus estimated SpO_2_, for conventional and designed sensor in specified range of oxygen saturation.

Sensor	Subject	<70	70∼80	80∼90	90∼100
Conventional sensor	Asian	−0.08 ± 2.24	−0.49 ± 2.38	1.39 ± 1.93	1.10 ± 1.87
	Caucasian	−1.59 ± 1.92	−0.45 ± 1.85	1.20 ± 1.92	1.29 ± 1.77
	African	−15.05 ± 5.58	−5.88 ± 5.08	−3.69 ± 3.98	−1.57 ± 2.25

Designed sensor	Asian	−1.83 ± 2.18	−0.95 ± 2.75	−0.78 ± 2.16	−0.79 ± 1.92
	Caucasian	−2.50 ± 0.71	0.74 ± 3.81	1.37 ± 3.36	0.04 ± 2.21
	African	−0.50 ± 2.86	0.98 ± 2.73	1.34 ± 1.92	0.68 ± 2.00

Data are presented as mean ± standard deviation.

## Data Availability

The data used to support the findings of this study are available from the corresponding author upon request.

## References

[1] Severinghaus J. W., Honda Y. (1987). History of blood gas analysis. VII. Pulse oximetry. *Journal of Clinical Monigoring*.

[2] Wukitsch M. W., Pettersono M. T., Tobler D. R., Pologe J. A. (1988). Pulse oximetry: analysis of theory, technology, and practice. *Journal of Clinical Monitoring*.

[3] Mendelson Y., Kent J. C., Yocum B. L., Birle M. J. (1988). Design and evaluation of a new reflectance pulse oximeter sensor. *Medical Instrumentation*.

[4] Johnson N., Johnson V. A., Fisher J., Jobbings B., Bannister J., Lilford R. J. (1991). Fetal monitoring with pulse oximetry. *BJOG: An International Journal of Obstetrics and Gynaecology*.

[5] König V., Huch R., Huch A. (1998). Reflectance pulse oximetry–principles and obstetric application in the zurich system. *Journal of Clinical Monitoring and Computing*.

[6] Lee H., Ko H., Lee J. (2016). Reflectance pulse oximetry: practical issues and limitations. *ICT Express*.

[7] Mendelson Y., Ochs B. D. (1988). Noninvasive pulse oximetry utilizing skin reflectace photoplethysmography. *IEEE Transactions on Biomedical Engineering*.

[8] Maxim Integrated (2018). MAX30100 pulse oximeter and heart-rate sensor IC for wearable health. https://datasheets.maximintegrated.com/en/ds/MAX30100.pdf.

[9] Brown W. E. L., Hill A. V. (1923). The oxygen-dissociation curve of blood, and its thermodynamical basis. *Proceedings of the Royal Society B: Biological Sciences*.

[10] Zhang H. F., Maslov K., Sivaramakrishnan M., Stoica G., Wang L. V. (2007). Imaging of hemoglobin oxygen saturation variations in single vessels in vivo using photoacustic microscopy. *Applied Physics Letters*.

[11] Bickler P. E., Feiner J. R., Severinghaus J. W. (2005). Effects of skin pigmentation on pulse oximeter accuracy at low saturation. *Anesthesiology*.

[12] Wang Y., Luo M. R., Wang M., Xiao K., Pointer M. (2017). Spectrophotometric measurement of human skin colour. *Color Research and Application*.

[13] Lu Z., Chen X., Dong Z., Zhao Z., Zhang X. (2016). A prototype of reflection pulse oximeter designed for mobile healthcare. *IEEE Journal of Biomedical and Health Informatics*.

[14] Poh M., Kim K., Goessling A., Swenson N., Picard R. (2012). Cardiovascular monitoring using earphones and a mobile device. *IEEE Pervasive Computing*.

[15] Venema B., Schiefer J., Blazek V., Blanik N., Leonhardt S. (2013). Evaluating innovative in-ear pulse oximetry for unobtsive cardiovascular and pulmonary monitoring during sleep. *IEEE Journal of Translational Engineering in Health and Medicine*.

[16] Wang W., den Brinker A. C., Stuikj S., de Haan G. (2017). Algorithmic principle of remote PPG. *IEEE Transactions on Biomedical Engineering*.

[17] Kumar M., Veeraraghavan A., Sabharwal A. (2015). DistancePPG: robust non-contact vital signs monitoring using a camera. *Biomedical Optics Express*.

[18] Coppetti T., Brauchlin A., Müggle S. (2017). Accuracy of smartphone apps for heart rate measurement. *European Journal of Preventive Cardiology*.

[19] Shao D., Liu C., Tsow F. (2016). Noncontact monitoring of blood oxygen saturation using camera and dual-wavelength imaging system. *IEEE Transactions on Biomedical Engineering*.

[20] Bal U. (2015). Non-contact estimation of heart rate and oxygen saturation using ambient light. *Biomedical Optics Express*.

[21] Tsai H.-Y., Huang K.-C., Chang H.-C., Chang C.-H. A study on oxygen saturation images constructed from the skin tissue of human hand.

[22] Tsai H.-Y., Huang K.-C., Chang H.-C., Yeh J.-L. A., Chang C.-H. (2014). A noncontact skin oxygen-saturation imaging system for measuring human tissue oxygen saturation. *IEEE Transactions on Instrumentation and Measurement*.

